# Supplementary figures and images for: Comparative characterization of flavivirus production in two cell lines: Human hepatoma-derived Huh7.5.1-8 and African green monkey kidney-derived Vero
Source: PLoS One. 2020 Apr 24;15(4):e0232274. doi: 10.1371/journal.pone.0232274 (PMC7182267; doi:10.1371/journal.pone.0232274)

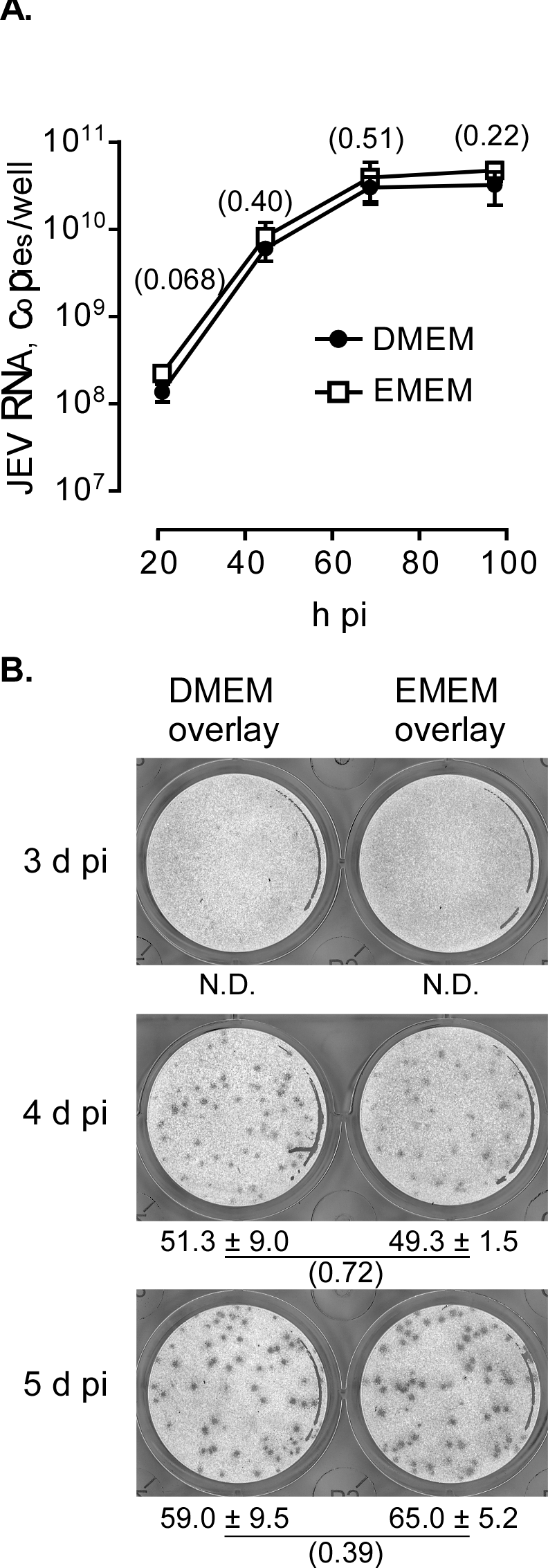

Supplement: S1 Fig — (A) Vero cells (No. JCRB9013) were seeded at 1 x 10^5^ cells per well of a 24-well plate one day before infection in DMEM supplemented with 10% (v/v) heat-inactivated FBS, 0.1 mM nonessential amino acids, 100 U/ml penicillin G, and 100 μg/ml streptomycin sulfate. After infection with JEV (Nakayama strain) at MOI 0.1, the cells were grown in the same medium (shown as closed circles) or EMEM-based medium as described in “Materials and methods” (shown as open squares). Culture supernatant was harvested at the indicated times, and RNA was extracted and purified to determine viral RNA copy number by qRT-PCR. Each point represents the mean ± SD of triplicates from one representative experiment. (B) Vero cells were infected with JEV and then incubated with 2.5 ml per well of DMEM overlay medium (DMEM supplemented with 0.2% [w/v] NaHCO3, 2% [v/v] heat-inactivated FBS, 2 mM L-alanyl-L-glutamine, 0.1 mM nonessential amino acids, and 1.25% [w/v] methylcellulose) or EMEM overlay medium (EMEM supplemented with 0.22% [w/v] NaHCO3, 2% [v/v] heat-inactivated FBS, 2 mM L-glutamine, and 1% [w/v] methylcellulose). The cells were fixed and stained at the indicated times. Values below the images are plaque numbers expressed as the mean ± SD of triplicates from one representative experiment. For both panels, statistical significance was determined by an unpaired two-tailed t test. Values in parentheses indicate p values, and those less than 0.05 were considered statistically significant. Similar results were obtained in another independent experiment. N.D., not detected. (TIF) [file pone.0232274.s001.tif]

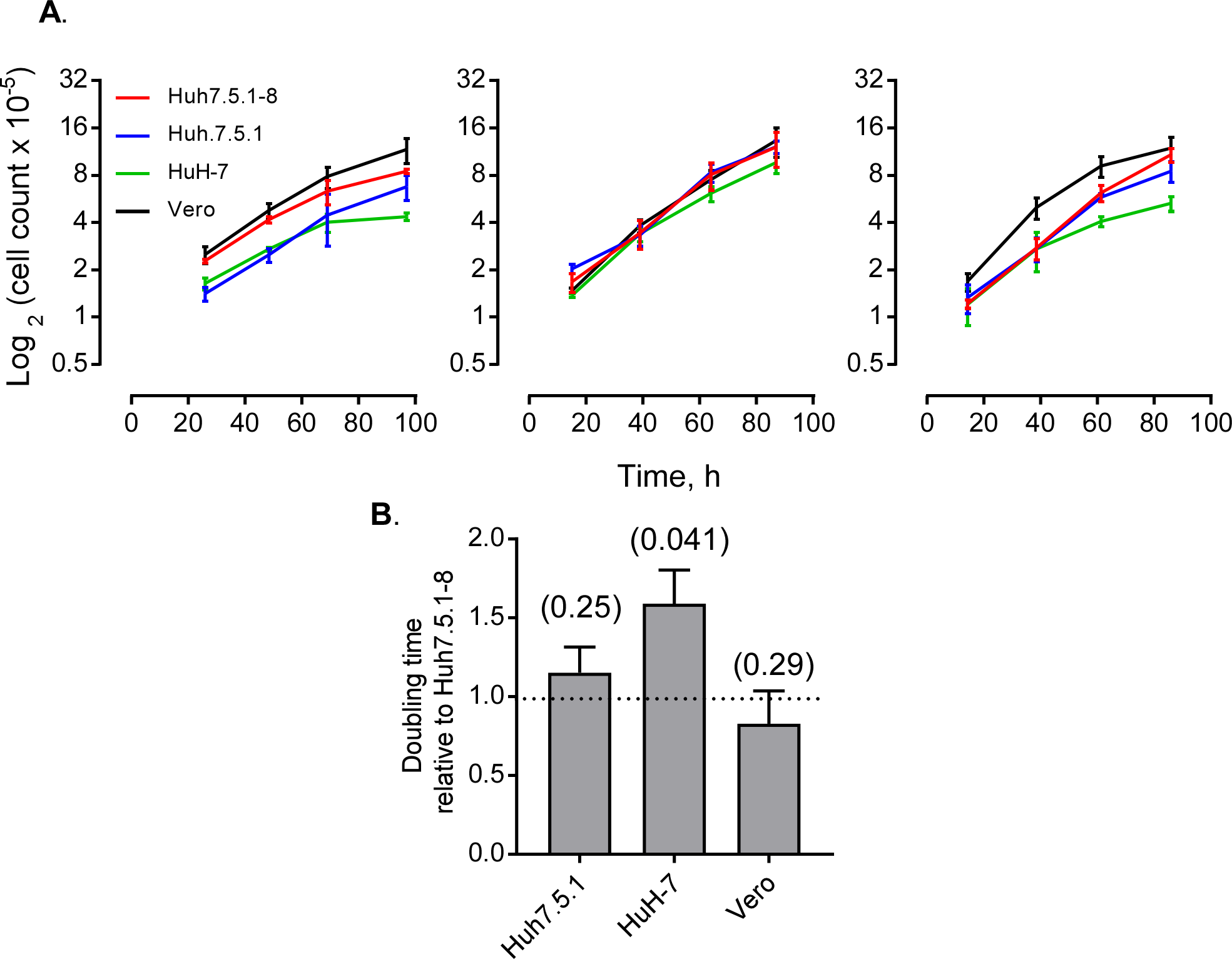

Supplement: S2 Fig — (A) Cells were seeded at 1 x 10^5^ cells per well of a 24-well plate. Cells were harvested at the indicated times and counted using the TC20 Automated Cell Counter (Bio-Rad). Results from three independent experiments are shown. (B) The doubling time of each cell line was calculated from the slope of the above graphs from day 1 to day 3 by linear regression analysis on GraphPad Prism (ver. 7.03) and then converted to a value relative to that of Huh7.5.1–8 cells. Bar with error bars represent the mean ± SD of the relative doubling times from three independent experiments. Statistical significance was determined by a one-sample t test with Bonferroni correction. Values in parentheses are p values, and those less than 0.0167 were considered statistically significant. (TIF) [file pone.0232274.s002.tif]

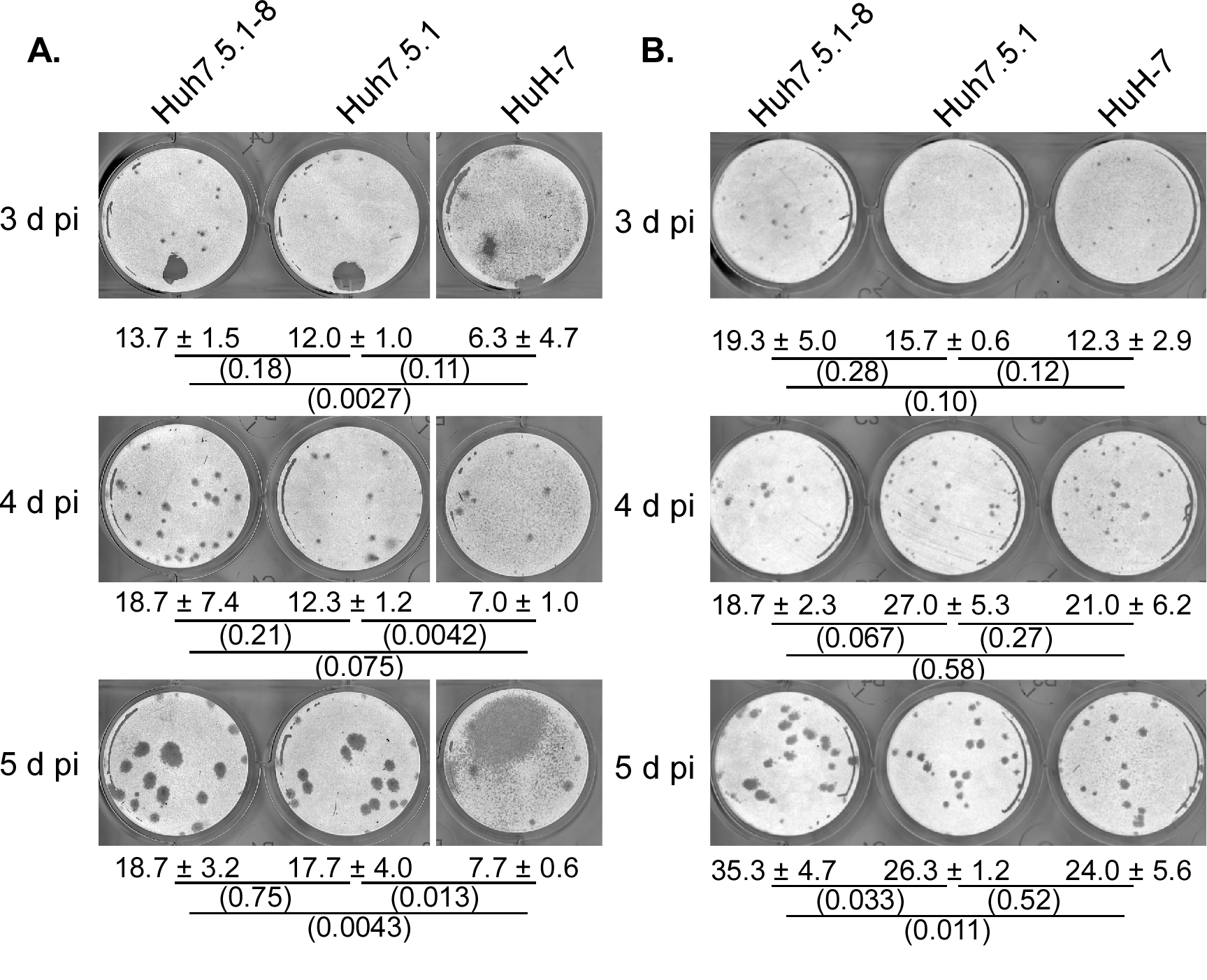

Supplement: S3 Fig — Cells were seeded at 4 x 10^5^ cells per well of a 12-well plate one day before infection and then infected with JEV at 40 PFU per well. The cells were fixed and stained at 3−5 d pi. Panels A and B represent different experiments. Values given below the images are plaque numbers expressed as the mean ± SD of triplicates from each experiment. Statistical significance was determined by an unpaired two-tailed t test with Bonferroni correction. Values in parentheses are p values, and those less than 0.0167 were considered statistically significant. (TIF) [file pone.0232274.s003.tif]

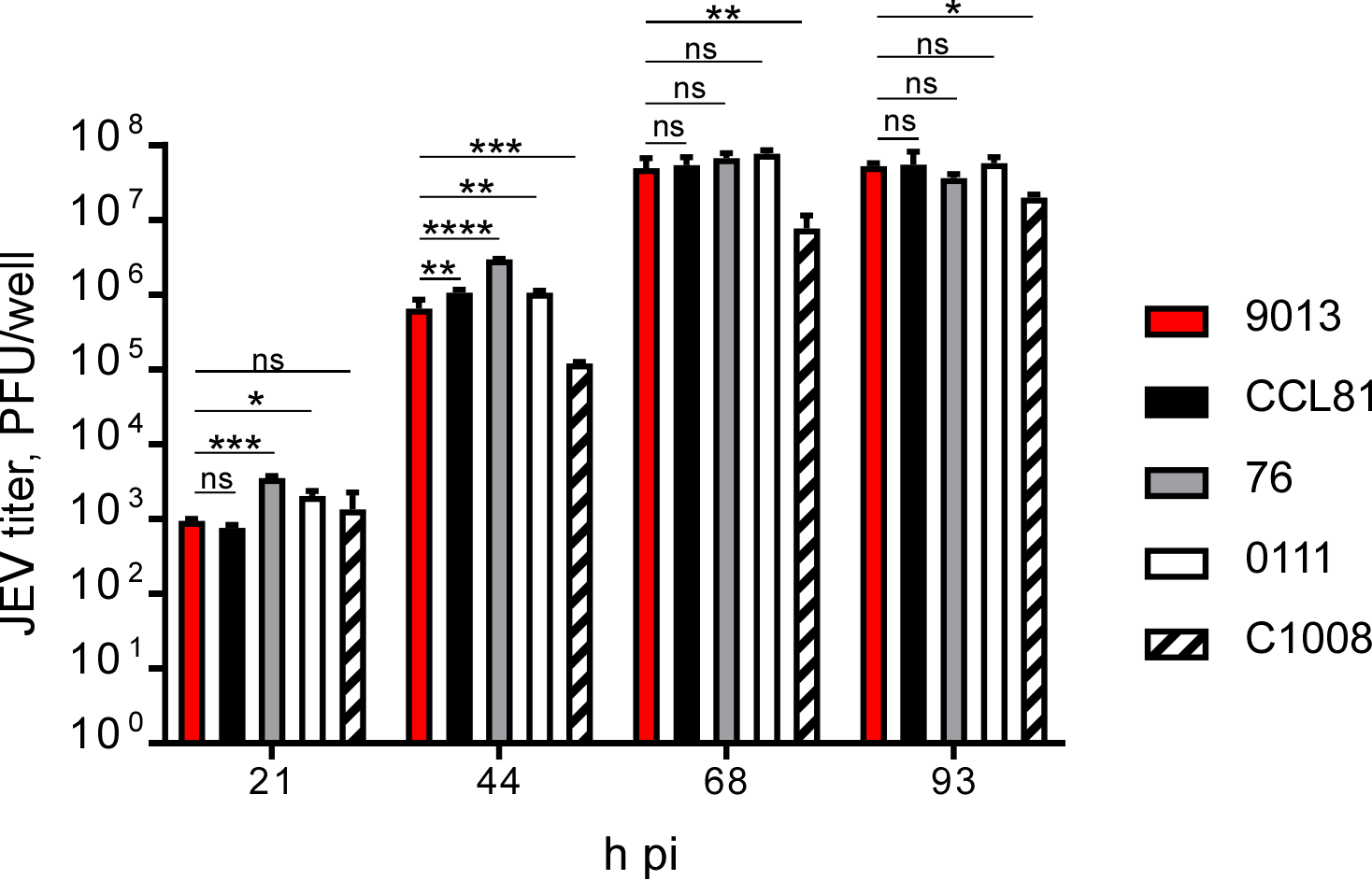

Supplement: S4 Fig — Vero ATCC CCL-81, Vero 76 (No. IFO50410), Vero 0111 (No. JCRB0111), and Vero C1008 (ATCC CRL-1586) cells were obtained from the National Institute of Biomedical Innovation (Osaka, Japan) as described (Sakuma C, Sekizuka T, Kuroda M, Kasai F, Saito K., Ikeda M, et al. Novel endogenous simian retroviral integrations in Vero cells: implications for quality control of a human vaccine cell substrate. Sci Rep, 2018; 8(1); 644. doi:10.1038/s41598-017-18934-2). Cells were seeded at 2 x 10^4^ cells per well of a 24-well plate one day before infection and then infected with JEV (Nakayama strain) at MOI 0.1. Culture supernatant was harvested at the indicated times to determine virus titers by plaque assay. Each point represents the mean ± SD of triplicates from one representative experiment. Statistical significance was determined by one-way ANOVA with Dunnett's multiple-comparison post-test. *, p < 0.05; **, p < 0.01; ***, p < 0.001; ****, p < 0.0001; ns, not significant. Similar results were obtained in another independent experiment. (TIF) [file pone.0232274.s004.tif]

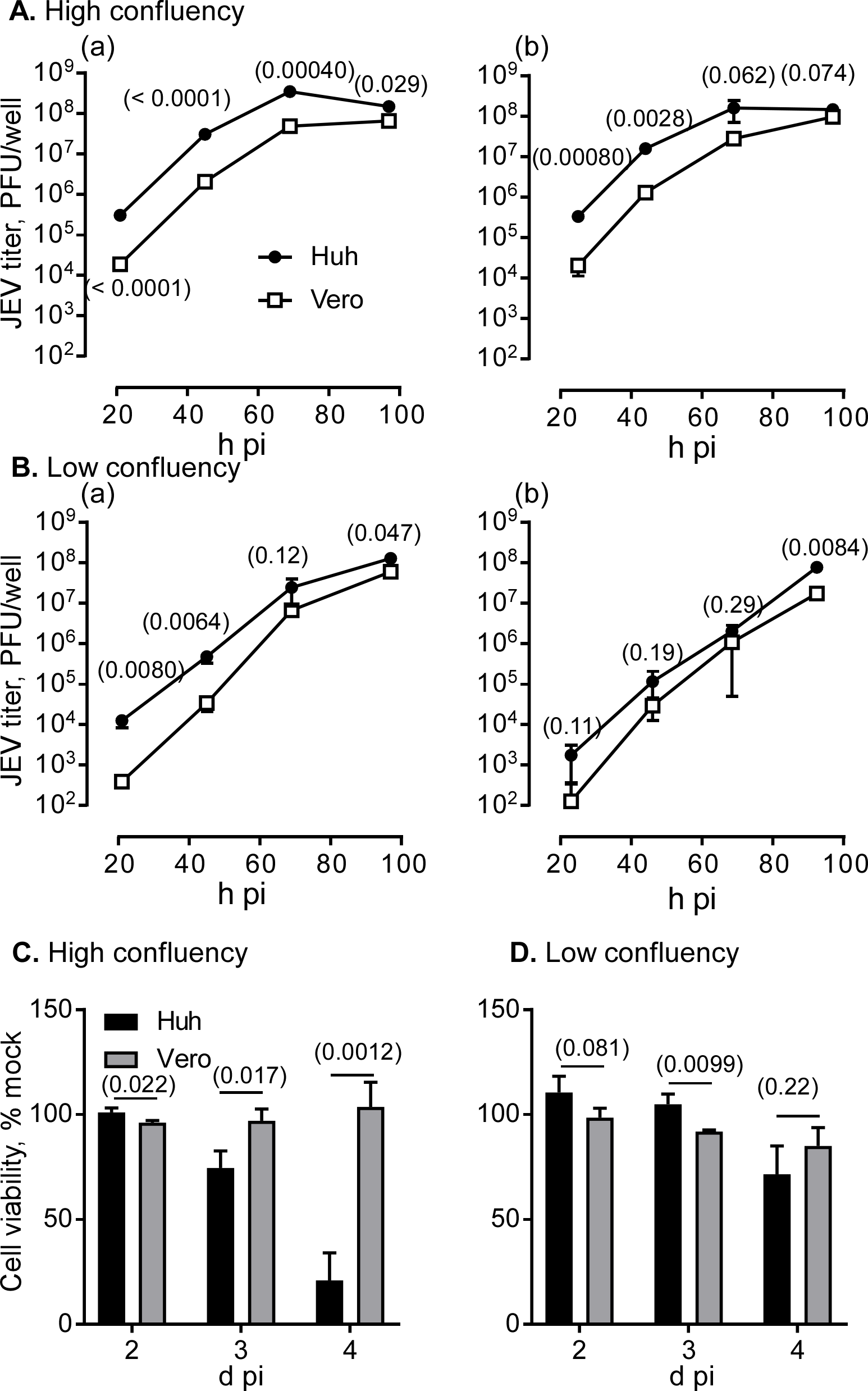

Supplement: S5 Fig — Cells were seeded and infected with JEV at MOI 0.01 under high (A, C) and low (B, D) confluency conditions as described in the legend to Fig 2. (A, B) Culture supernatants were harvested at the indicated times, and virus titers in the supernatants were determined by plaque assay. Results from two independent experiments (a, b) are shown. (C, D) Cell viability was determined at the indicated times and expressed as % of viability of mock-infected cells. Bars with error bars represent the mean ± SD of three independent experiments. For all panels, statistical significance was determined by an unpaired two-tailed t test. Values in parentheses are p values, and those less than 0.05 were considered statistically significant. Huh, Huh7.5.1–8 cells. (TIF) [file pone.0232274.s005.tif]

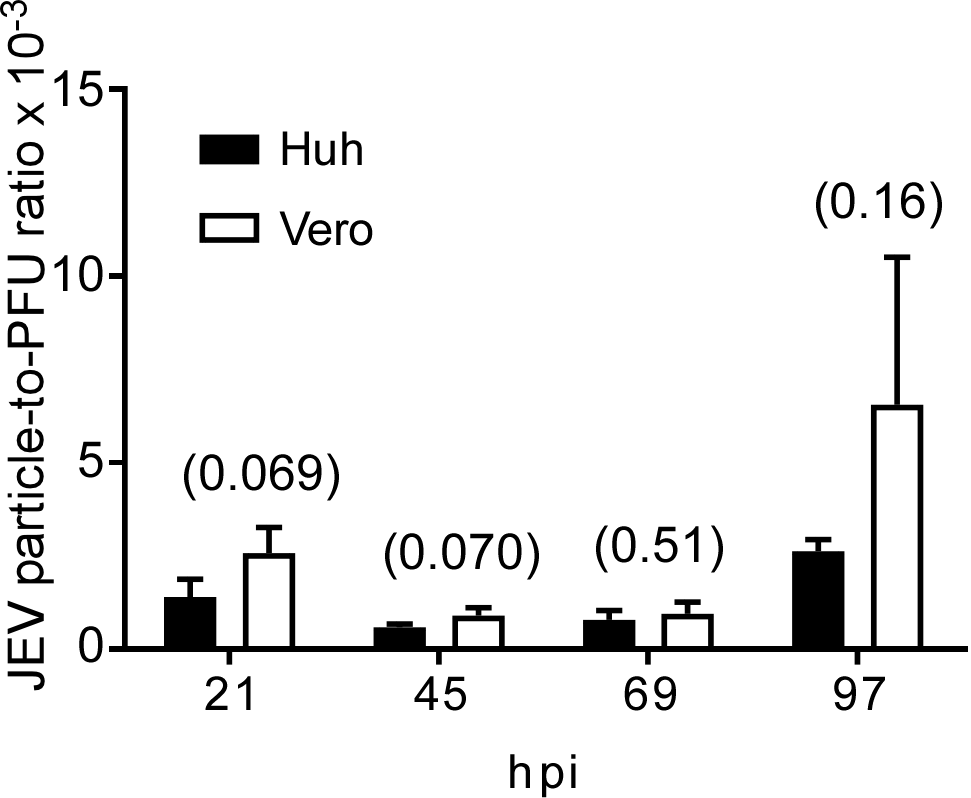

Supplement: S6 Fig — Cells were infected with JEV at MOI 0.1 under high confluency conditions as described in the legend to Fig 2, and the culture supernatants were harvested at the indicated times. RNA was extracted and purified from each culture supernatant, and viral RNA copy number in the supernatant was determined by qRT-PCR. A virus titer in PFU of each culture supernatant was also determined by plaque assay, and particle-to-PFU ratio was calculated. Each point represents the mean ± SD of triplicates from one representative experiment. Statistical significance was determined by an unpaired two-tailed t test. Values in parentheses indicate p values, and those less than 0.05 were considered statistically significant. Huh, Huh7.5.1–8 cells. (TIF) [file pone.0232274.s006.tif]

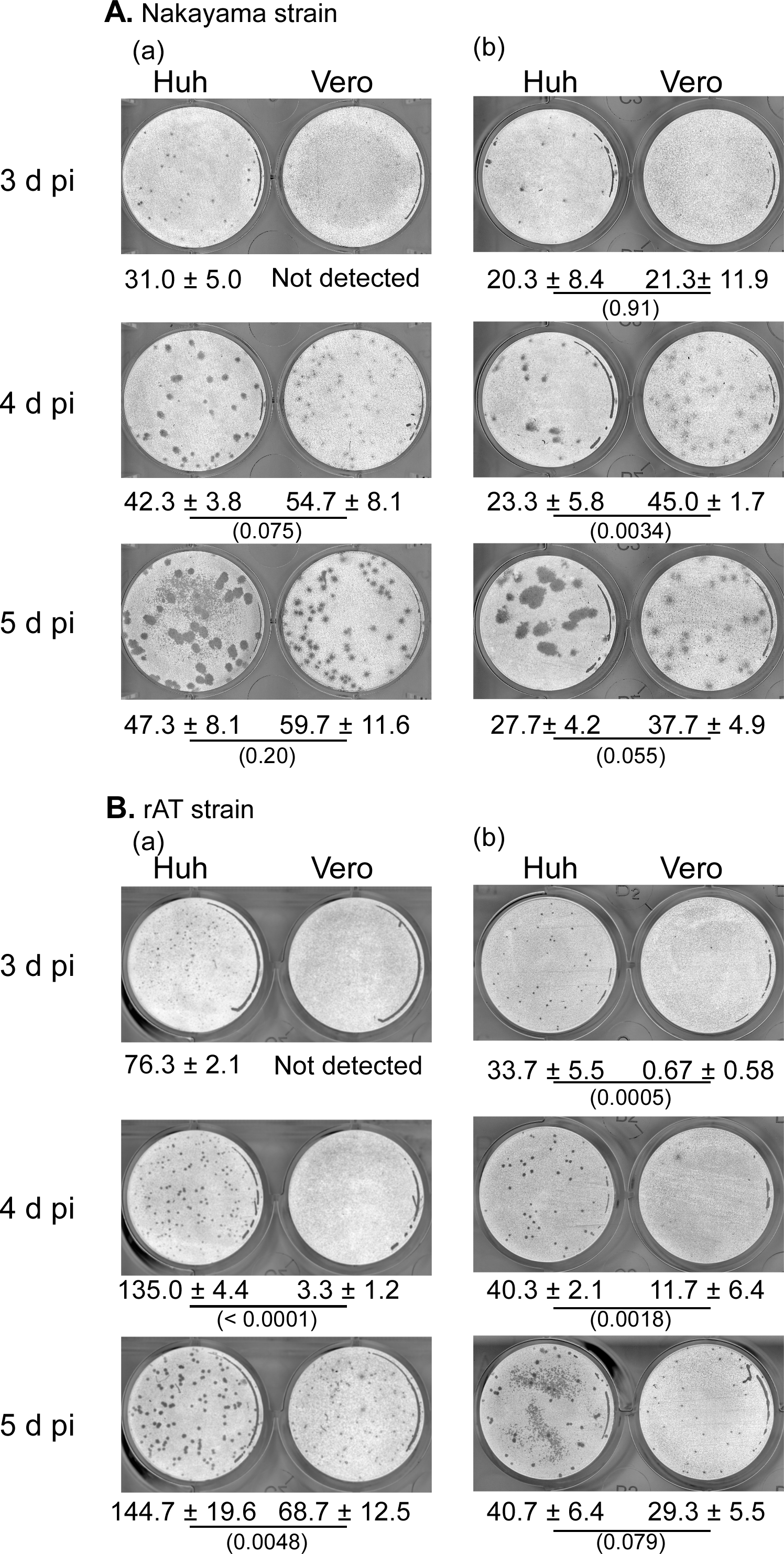

Supplement: S7 Fig — Representative black-and-white inverted images of plaques of the JEV Nakayama strain (A) and the rAT strain (B) in Huh7.5.1–8 (Huh) and Vero cells. Cells were fixed and stained at the indicated times. Values given below the images indicate plaque numbers expressed as the mean ± SD of triplicates from one representative experiment. Results from two independent experiments (a, b) are shown. Statistical significance was determined by an unpaired two-tailed t test. Values in parentheses indicate p values, and those less than 0.05 were considered statistically significant. (TIF) [file pone.0232274.s007.tif]

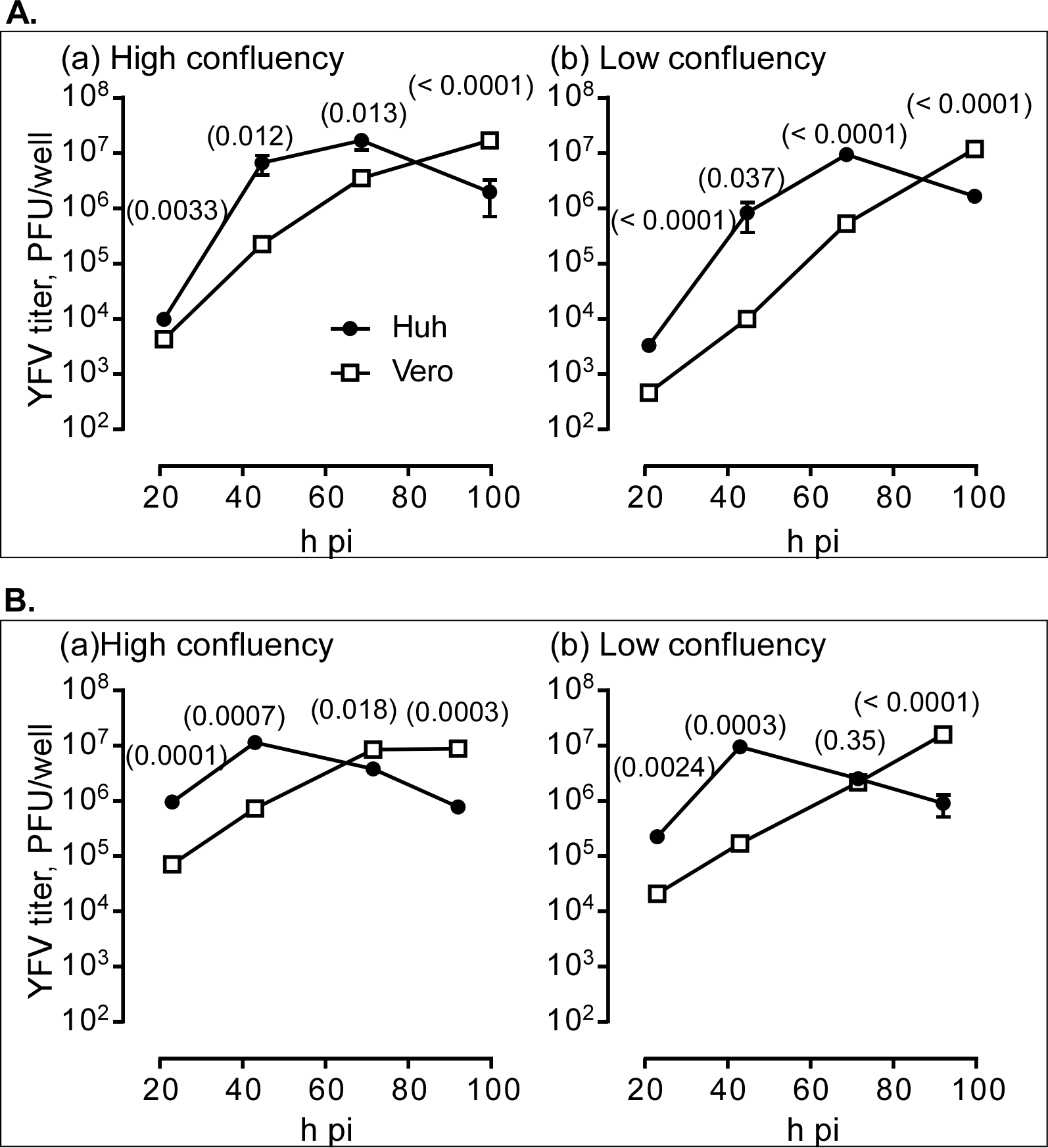

Supplement: S8 Fig — Cells were seeded and infected with YFV at MOI 0.1 under high (a) and low (b) confluency conditions as described in the legend to Fig 2. (A, B) Culture supernatants were harvested at the indicated times, and virus titers in the supernatants were determined by plaque assay. Results from two independent experiments (A, B) are shown. Statistical significance was determined by an unpaired two-tailed t test. Values in parentheses are p values, and those less than 0.05 were considered statistically significant. Huh, Huh7.5.1–8 cells. (TIF) [file pone.0232274.s008.tif]

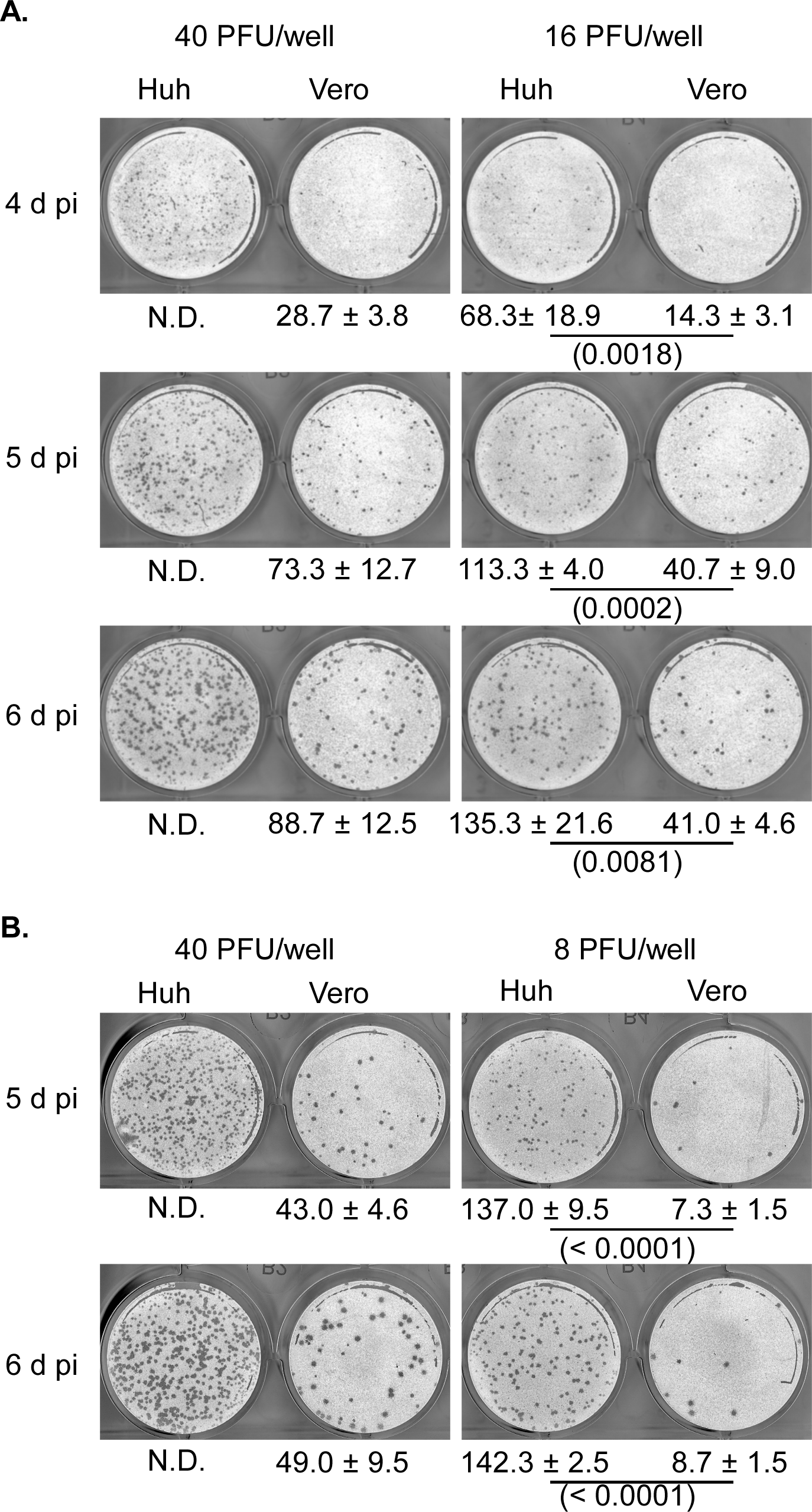

Supplement: S9 Fig — Representative black-and-white inverted images of YFV plaques in Huh7.5.1–8 (Huh) and Vero cells. Cells were infected with the indicated amount (PFU) of YFV (17D-204 strain) per well and then fixed and stained at the indicated times. Results from two independent experiments (A, B) are shown. Values below the images indicate plaque numbers expressed as the mean ± SD of triplicates from one representative experiment. Statistical significance was determined by an unpaired two-tailed t test. Values in parentheses indicate p values, and those less than 0.05 were considered statistically significant. N.D., not determined. (TIF) [file pone.0232274.s009.tif]
