# Supplementary material for: Comparative characterization of flavivirus production in two cell lines: Human hepatoma-derived Huh7.5.1-8 and African green monkey kidney-derived Vero
Source: PLoS One. 2020 Apr 24;15(4):e0232274. doi: 10.1371/journal.pone.0232274 (PMC7182267; doi:10.1371/journal.pone.0232274)
Supplement: S10 Fig — Alignment of nucleotide sequences was performed using GENETYX version 14.0.0 (GENETYX Co., Tokyo, Japan). (PDF) [file pone.0232274.s010.pdf]

S10 Fig. Nucleotide sequences of RIG-I full-length cDNAs (FL1,2) and splice variants (SV1, 2).

```
AF038963.1    1 TAGTTATTAAAGTTCCTATGCAGCTCCGCCTCCGTCCGGCCTCATTTCCTCAAAAAATCC  60
FL1           1 ---------------------------------GTCCGGCCTCATTTCCTCGGAAAATCC  27
FL2           1 ---------------------------------GTCCGGCCTCATTTCCTCGGAAAATCC  27
SV1           1 ---------------------------------GTCCGGCCTCATTTCCTCGGAAAATCC  27
SV2           1 ---------------------------------GTCCGGCCTCATTTCCTCGGAAAATCC  27

AF038963.1   61 CTGCTTTCCCCGCTCGCCACGCCCTCCTGCTACCCGGCTTTAAAGCTAGTGAGGCACAGC 120
FL1          28 CTGCTTTCCCCGCTCGCCACGCCCTCCTGCTACCCGGCTTTAAAGCTAGTGAGGCACAGC  87
FL2          28 CTGCTTTCCCCGCTCGCCACGCCCTCCTGCTACCCGGCTTTAAAGCTAGTGAGGCACAGC  87
SV1          28 CTGCTTTCCCCGCTCGCCACGCCCTCCTGCTACCCGGCTTTAAAGCTAGTGAGGCACAGC  87
SV2          28 CTGCTTTCCCCGCTCGCCACGCCCTCCTGCTACCCGGCTTTAAAGCTAGTGAGGCACAGC  87

AF038963.1  121 CTGCGGGGAACGTAGCTAGCTGCAAGCAGAGGCCGGCATGACCACCGAGCAGCGACGCAG 180
FL1          88 CTGCGGGGAACGTAGCTAGCTGCAAGCAGAGGCCGGCATGACCACCGAGCAGCGACGCAG 147
FL2          88 CTGCGGGGAACGTAGCTAGCTGCAAGCAGAGGCCGGCATGACCACCGAGCAGCGACGCAG 147
SV1          88 CTGCGGGGAACGTAGCTAGCTGCAAGCAGAGGCCGGCATGACCACCGAGCAGCGACGCAG 147
SV2          88 CTGCGGGGAACGTAGCTAGCTGCAAGCAGAGGCCGGCATGACCACCGAGCAGCGACGCAG 147

AF038963.1  181 CCTGCAAGCCTTCCAGGATTATATCCGGAAGACCCTGGACCCTACCTACATCCTGAGCTA 240
FL1         148 CCTGCAAGCCTTCCAGGATTATATCCGGAAGACCCTGGACCCTACCTACATCCTGAGCTA 207
FL2         148 CCTGCAAGCCTTCCAGGATTATATCCGGAAGACCCTGGACCCTACCTACATCCTGAGCTA 207
SV1         148 CCTGCAAGCCTTCCAGGATTATATCCGGAAGACCCTGGACCCTACCTACATCCTGAGCTA 207
SV2         148 CCTGCAAGCCTTCCAGGATTATATCCGGAAGACCCTGGACCCTACCTACATCCTGAGCTA 207

AF038963.1  241 CATGGCCCCCTGGTTTAGGGAGGAAGAGGTGCAGTATATTCAGGCTGAGAAAAACAACAA 300
FL1         208 CATGGCCCCCTGGTTTAGGGAGGAAGAGGTGCAGTATATTCAGGCTGAGAAAAACAACAA 267
FL2         208 CATGGCCCCCTGGTTTAGGGAGGAAGAGGTGCAGTATATTCAGGCTGAGAAAAACAACAA 267
SV1         208 CATGGCCCCCTGGTTTAGGGAGGAAGAGGTGCAGTATATTCAGGCTGAGAAAAACAACAA 267
SV2         208 CATGGCCCCCTGGTTTAGGGAGGAAGAGGTGCAGTATATTCAGGCTGAGAAAAACAACAA 267

AF038963.1  301 GGGCCCAATGGAGGCTGCCACACTTTTTCTCAAGTTCCTGTTGGAGCTCCAGGAGGAAGG 360
FL1         268 GGGCCCAATGGAGGCTGCCACACTTTTTCTCAAGTTCCTGTTGGAGCTCCAGGAGGAAGG 327
FL2         268 GGGCCCAATGGAGGCTGCCATACTTTTTCTCAAGTTCCTGTTGGAGCTCCAGGAGGAAGG 327
SV1         268 GGGCCCAATGGAGGCTGCCACACTTTTTCTCAAGTTCCTGTTGGAGCTCCAGGAGGAAGG 327
SV2         268 GGGCCCAATGGAGGCTGCCACACTTTTTCTCAAGTTCCTGTTGGAGCTCCAGGAGGAAGG 327

AF038963.1  361 CTGGTTCCGTGGCTTTTTGGATGCCCTAGACCATGCAGGTTATTCTGGACTTTATGAAGC 420
FL1         328 CTGGTTCCGTGGCTTTTTGGATGCCCTAGACCATGCAGGTTATTCTGGACTTTATGAAGC 387
FL2         328 CTGGTTCCGTGGCTTTTTGGATGCCCTAGACCATGCAGGTTATTCTGGACTTTATGAAGC 387
SV1         328 CTGGTTCCGTGGCTTTTTGGATGCCCTAGACCATGCAGGTTATTCTGGACTTTATGAAGC 387
SV2         328 CTGGTTCCGTGGCTTTTTGGATGCCCTAGACCATGCAGGTTATTCTGGACTTTATGAAGC 387

AF038963.1  421 CATTGAAAGTTGGGATTTCAAAAAAATTGAAAAGTTGGAGGAGTATAGATTACTTTTAAA 480
FL1         388 CATTGAAAGTTGGGATTTCAAAAAAATTGAAAAGTTGGAGGAGTATAGATTACTTTTAAA 447
FL2         388 CATTGAAAGTTGGGATTTCAAAAAAATTGAAAAGTTGGAGGAGTATAGATTACTTTTAAA 447
SV1         388 CATTGAAAGTTGGGATTTCAAAAAAATTGAAAAGTTGGAGGAGTATAGATTACTTTTAAA 447
SV2         388 CATTGAAAGTTGGGATTTCAAAAAAATTGAAAAGTTGGAGGAGTATAGATTACTTTTAAA 447

AF038963.1  481 ACGTTTACAACCAGAATTTAAAACCAGAATTATCCCAACCGATATCATTTCTGATCTGTC 540
FL1         448 ACGTTTACAACCAGAATTTAAAACCAGAATTATCCCAACCGATATCATTTCTGATCTGTC 507
FL2         448 ACGTTTACAACCAGAATTTAAAACCAGAATTATCCCAACCGATATCATTTCTGATCTGTC 507
SV1         448 ACGTTTACAACCAGAATTTAAAACCAGAATTATCCCAACCGATATCATTTCTGATCTGTC 507
SV2         448 ACGTTTACAACCAGAATTTAAAACCAGAATTATCCCAACCGATATCATTTCTGATCTGTC 507

AF038963.1  541 TGAATGTTTAATTAATCAGGAATGTGAAGAAATTCTACAGATTTGCTCTACTAAGGGGAT 600
FL1         508 TGAATGTTTAATTAATCAGGAATGTGAAGAAATTCTACAGATTTGCTCTACTAAGGGGAT 567
FL2         508 TGAATGTTTAATTAATCAGGAATGTGAAGAAATTCTACAGATTTGCTCTACTAAGGGGAT 567
SV1         508 TGAATGTTTAATTAATCAGGAATGTGAAGAAATTCTACAGATTTGCTCTACTAAGGGGAT 567
SV2         508 TGAATGTTTAATTAATCAGGAATGTGAAGAAATTCTACAGATTTGCTCTACTAAGGGGAT 567

AF038963.1  601 GATGGCAGGTGCAGAGAAATTGGTGGAATGCCTTCTCAGATCAGACAAGGAAAACTGGCC 660
FL1         568 GATGGCAGGTGCAGAGAAATTGGTGGAATGCCTTCTCAGATCAGACAAGGAAAACTGGCC 627
FL2         568 GATGGCAGGTGCAGAGAAATTGGTGGAATGCCTTCTCAGATCAGACAAGGAAAACTGGCC 627
SV1         568 GATGGCAGGTGCAGAGAAATTGGTGGAATGCCTTCTCAGATCAGACAAGGAAAACTGGCC 627
SV2         568 GATGGCAGGTGCAGAGAAATTGGTGGAATGCCTTCTCAGATCAGACAAGGAAAACTGGCC 627
```

```
AF038963.1  661 CAAAACTTTGAAACTTGCTTTGGAGAAAGAAAGGAACAAGTTCAGTGAACTGTGGATTGT 720
FL1         628 CAAAACTTTGAAACTTGCTTTGGAGAAAGAAAGGAACAAGTTCAGTGAACTGTGGATTGT 687
FL2         628 CAAAACTTTGAAACTTGCTTTGGAGAAAGAAAGGAACAAGTTCAGTGAACTGTGGATTGT 687
SV1         628 CAAAACTTTGAAACTTGCTTTGGAGAAAGAAAGGAACAAGTTCAGTGAACTGTGGATTGT 687
SV2         628 CAAAACTTTGAAACTTGCTTTGGAGAAAGAAAGGAACAAGTTCAGTGAACTGTGGATTGT 687

AF038963.1  721 AGAGAAAGGTATAAAAGATGTTGAAACAGAAGATCTTGAGGATAAGATGGAAACTTCTGA 780
FL1         688 AGAGAAAGGTATAAAAGATGTTGAAACAGAAGATCTTGAGGATAAGATGGAAACTTCTGA 747
FL2         688 AGAGAAAGGTATAAAAGATGTTGAAACAGAAGATCTTGAGGATAAGATGGAAACTTCTGA 747
SV1         688 AGAGAAAGGTATAAAAGATGTTGAAACAGAAGATCTTGAGGATAAGATGGAAACTTCTGA 747
SV2         688 AGAGA------------------------------------------------------- 692

AF038963.1  781 CATACAGATTTTCTACCAAGAAGATCCAGAATGCCAGAATCTTAGTGAGAATTCATGTCC 840
FL1         748 CATACAGATTTTCTACCAAGAAGATCCAGAATGCCAGAATCTTAGTGAGAATTCATGTCC 807
FL2         748 CATACAGATTTTCTACCAAGAAGATCCAGAATGCCAGAATCTTAGTGAGAATTCATGTCC 807
SV1         748 CATACAGATTTTCTACCAAGAAGATCCAGAATGCCAGAATCTTAGTGAGAATTCATGTCC 807
SV2         692 ------------------------------------------------------------ 692

AF038963.1  841 ACCTTCAGAAGTGTCTGATACAAACTTGTACAGCCCATTTAAACCAAGAAATTACCAATT 900
FL1         808 ACCTTCAGAAGTGTCTGATACAAACTTGTACAGCCCATTTAAACCAAGAAATTACCAATT 867
FL2         808 ACCTTCAGAAGTGTCTGATACAAACTTGTACAGCCCATTTAAACCAAGAAATTACCAATT 867
SV1         808 ACCTTC------------------------------------------------------ 813
SV2         692 ------------------------------------------------------------ 692

AF038963.1  901 AGAGCTTGCTTTGCCTGCTATGAAAGGAAAAAACACAATAATATGTGCTCCTACAGGTTG 960
FL1         868 AGAGCTTGCTTTGCCTGCTATGAAAGGAAAAAACACAATAATATGTGCTCCTACAGGTTG 927
FL2         868 AGAGCTTGCTTTGCCTGCTATGAAAGGAAAAAACACAATAATATGTGCTCCTACAGGTTG 927
SV1         813 ------------------------------------------------------------ 813
SV2         692 ------------------------------------------------------------ 692

AF038963.1  961 TGGAAAAACCTTTGTTTCACTGCTTATATGTGAACATCATCTTAAAAAATTCCCACAAGG 1020
FL1         928 TGGAAAAACCTTTGTTTCACTGCTTATATGTGAACATCATCTTAAAAAATTCCCACAAGG 987
FL2         928 TGGAAAAACCTTTGTTTCACTGCTTATATGTGAACATCATCTTAAAAAATTCCCACAAGG 987
SV1         813 ------------------------------------------------------------ 813
SV2         692 ------------------------------------------------------------ 692

AF038963.1 1021 ACAAAAGGGGAAAGTTGTCTTTTTTGCGAATCAGATCCCAGTGTATGAACAGCAGAAATC 1080
FL1         988 ACAAAAGGGGAAAGTTGTCTTTTTTGCGAATCAGATCCCAGTGTATGAACAGCAGAAATC 1047
FL2         988 ACAAAAGGGGAAAGTTGTCTTTTTTGCGAATCAGATCCCAGTGTATGAACAGCAGAAATC 1047
SV1         813 ------------------------------------------------------------ 813
SV2         692 ------------------------------------------------------------ 692

AF038963.1 1081 TGTATTCTCAAAATACTTTGAAAGACATGGGTATAGAGTTACAGGCATTTCTGGAGCAAC 1140
FL1        1048 TGTATTCTCAAAATACTTTGAAAGACATGGGTATAGAGTTACAGGCATTTCTGGAGCAAC 1107
FL2        1048 TGTATTCTCAAAATACTTTGAAAGACATGGGTATAGAGTTACAGGCATTTCTGGAGCAAC 1107
SV1         813 ------------------------------------------------------------ 813
SV2         692 ------------------------------------------------------------ 692

AF038963.1 1141 AGCTGAGAATGTCCCAGTGGAACAGATTGTTGAGAACAATGACATCATCATTTTAACTCC 1200
FL1        1108 AGCTGAGAATGTCCCAGTGGAACAGATTGTTGAGAACAATGACATCATCATTTTAACTCC 1167
FL2        1108 AGCTGAGAATGTCCCAGTGGAACAGATTGTTGAGAACAATGACATCATCATTTTAACTCC 1167
SV1         813 ------------------------------------------------------------ 813
SV2         692 ------------------------------------------------------------ 692

AF038963.1 1201 ACAGATTCTTGTGAACAACCTTAAAAAGGGAACGATTCCATCACTATCCATCTTTACTTT 1260
FL1        1168 ACAGATTCTTGTGAACAACCTTAAAAAGGGAACGATTCCATCACTATCCATCTTTACTTT 1227
FL2        1168 ACAGATTCTTGTGAACAACCTTAAAAAGGGAACGATTCCATCACTATCCATCTTTACTTT 1227
SV1         813 ------------------------------------------------------------ 813
SV2         692 ------------------------------------------------------------ 692

AF038963.1 1261 GATGATATTTGATGAATGCCACAACACTAGTAAACAACACCCGTACAATATGATCATGTT 1320
FL1        1228 GATGATATTTGATGAATGCCACAACACTAGTAAACAACACCCGTACAATATGATCATGTT 1287
FL2        1228 GATGATATTTGATGAATGCCACAACACTAGTAAACAACACCCGTACAATATGATCATGTT 1287
SV1         813 ------------------------------------------------------------ 813
SV2         692 ------------------------------------------------------------ 692
```

```
AF038963.1 1321 TAATTATCTAGATCAGAAACTTGGAGGATCTTCAGGCCCACTGCCCCAGGTCATTGGGCT 1380
FL1        1288 TAATTATCTAGATCAGAAACTTGGAGGATCTTCAGGCCCACTGCCCCAGGTCATTGGGCT 1347
FL2        1288 TAATTATCTAGATCAGAAACTTGGAGGATCTTCAGGCCCACTGCCCCAGGTCATTGGGCT 1347
SV1         813 ------------------------------------------------------------ 813
SV2         692 ------------------------------------------------------------ 692

AF038963.1 1381 GACTGCCTCGGTTGGTGTTGGGGATGCCAAAAACACAGATGAAGCCTTGGATTATATCTG 1440
FL1        1348 GACTGCCTCGGTTGGTGTTGGGGATGCCAAAAACACAGATGAAGCCTTGGATTATATCTG 1407
FL2        1348 GACTGCCTCGGTTGGTGTTGGGGATGCCAAAAACACAGATGAAGCCTTGGATTATATCTG 1407
SV1         813 ------------------------------------------------------------ 813
SV2         692 ------------------------------------------------------------ 692

AF038963.1 1441 CAAGCTGTGTGCTTCTCTTGATGCGTCAGTGATAGCAACAGTCAAACACAATCTGGAGGA 1500
FL1        1408 CAAGCTGTGTGCTTCTCTTGATGCGTCAGTGATAGCAACAGTCAAACACAATCTGGAGGA 1467
FL2        1408 CAAGCTGTGTGCTTCTCTTGATGCGTCAGTGATAGCAACAGTCAAACACAATCTGGAGGA 1467
SV1         813 ------------------------------------------------------------ 813
SV2         692 ------------------------------------------------------------ 692

AF038963.1 1501 ACTGGAGCAAGTTGTTTATAAGCCCCAGAAGTTTTTCAGGAAAGTGGAATCACGGATTAG 1560
FL1        1468 ACTGGAGCAAGTTGTTTATAAGCCCCAGAAGTTTTTCAGGAAAGTGGAATCACGGATTAG 1527
FL2        1468 ACTGGAGCAAGTTGTTTATAAGCCCCAGAAGTTTTTCAGGAAAGTGGAATCACGGATTAG 1527
SV1         813 ------------------------------------------------------------ 813
SV2         692 ------------------------------------------------------------ 692

AF038963.1 1561 CGACAAATTTAAATACATCATAGCTCAGCTGATGAGGGACACAGAGAGTCTGGCAAAGAG 1620
FL1        1528 CGACAAATTTAAATACATCATAGCTCAGCTGATGAGGGACACAGAGAGTCTGGCAAAGAG 1587
FL2        1528 CGACAAATTTAAATACATCATAGCTCAGCTGATGAGGGACACAGAGAGTCTGGCAAAGAG 1587
SV1         813 ------------------------------------------------------------ 813
SV2         692 ------------------------------------------------------------ 692

AF038963.1 1621 AATCTGCAAAGACCTCGAAAACTTATCTCAAATTCAAAATAGGGAATTTGGAACACAGAA 1680
FL1        1588 AATCTGCAAAGACCTCGAAAACTTATCTCAAATTCAAAATAGGGAATTTGGAACACAGAA 1647
FL2        1588 AATCTGCAAAGACCTCGAAAACTTATCTCAAATTCAAAATAGGGAATTTGGAACACAGAA 1647
SV1         813 ------------------------------------------------------------ 813
SV2         692 ------------------------------------------------------------ 692

AF038963.1 1681 ATATGAACAATGGATTGTTACAGTTCAGAAAGCATGCATGGTGTTCCAGATGCCAGACAA 1740
FL1        1648 ATATGAACAATGGATTGTTACAGTTCAGAAAGCATGCATGGTGTTCCAGATGCCAGACAA 1707
FL2        1648 ATATGAACAATGGATTGTTACAGTTCAGAAAGCATGCATGGTGTTCCAGATGCCAGACAA 1707
SV1         813 ------------------------------------------------------------ 813
SV2         692 ------------------------------------------------------------ 692

AF038963.1 1741 AGATGAAGAGAGCAGGATTTGTAAAGCCCTGTTTTTATACACTTCACATTTGCGGAAATA 1800
FL1        1708 AGATGAAGAGAGCAGGATTTGTAAAGCCCTGTTTTTATACACTTCACATTTGCGGAAATA 1767
FL2        1708 AGATGAAGAGAGCAGGATTTGTAAAGCCCTGTTTTTATACACTTCACATTTGCGGAAATA 1767
SV1         813 ------------------------------------------------------------ 813
SV2         692 ------------------------------------------------------------ 692

AF038963.1 1801 TAATGATGCCCTCATTATCAGTGAGCATGCACGAATGAAAGATGCTCTGGATTACTTGAA 1860
FL1        1768 TAATGATGCCCTCATTATCAGTGAGCATGCACGAATGAAAGATGCTCTGGATTACTTGAA 1827
FL2        1768 TAATGATGCCCTCATTATCAGTGAGCATGCACGAATGAAAGATGCTCTGGATTACTTGAA 1827
SV1         813 ------------------------------------------------------------ 813
SV2         692 ------------------------------------------------------------ 692

AF038963.1 1861 AGACTTCTTCAGCAATGTCCGAGCAGCAGGATTCGAAGAGATTGAGCAAGATCTTACTCA 1920
FL1        1828 AGACTTCTTCAGCAATGTCCGAGCAGCAGGATTCGATGAGATTGAGCAAGATCTTACTCA 1887
FL2        1828 AGACTTCTTCAGCAATGTCCGAGCAGCAGGATTCGATGAGATTGAGCAAGATCTTACTCA 1887
SV1         813 ------------------------------------------------------------ 813
SV2         692 ------------------------------------------------------------ 692

AF038963.1 1921 GAGATTTGAAGAAAAGCTGCAGGAACTAGAAAGTGTTTCCAGGGATCCCAGCAATGAGAA 1980
FL1        1888 GAGATTTGAAGAAAAGCTGCAGGAACTAGAAAGTGTTTCCAGGGATCCCAGCAATGAGAA 1947
FL2        1888 GAGATTTGAAGAAAAGCTGCAGGAACTAGAAAGTGTTTCCAGGGATCCCAGCAATGAGAA 1947
SV1         814 ---------AGAAAAGCTGCAGGAACTAGAAAGTGTTTCCAGGGATCCCAGCAATGAGAA 864
SV2         693 --------AAGAAAAGCTGCAGGAACTAGAAAGTGTTTCCAGGGATCCCAGCAATGAGAA 744
```

```
AF038963.1 1981 TCCTAAACTTGAAGACCTCTGCTTCATCTTACAAGAAGAGTACCACTTAAACCCAGAGAC 2040
FL1        1948 TCCTAAACTTGAAGACCTCTGCTTCATCTTACAAGAAGAGTACCACTTAAACCCAGAGAC 2007
FL2        1948 TCCTAAACTTGAAGACCTCTGCTTCATCTTACAAGAAGAGTACCACTTAAACCCAGAGAC 2007
SV1         865 TCCTAAACTTGAAGACCTCTGCTTCATCTTACAAGAAGAGTACCACTTAAACCCAGAGAC 924
SV2         745 TCCTAAACTTGAAGACCTCTGCTTCATCTTACAAGAAGAGTACCACTTAAACCCAGAGAC 804

AF038963.1 2041 AATAACAATTCTCTTTGTGAAAACCAGAGCACTTGTGGACGCTTTAAAAAATTGGATTGA 2100
FL1        2008 AATAACAATTCTCTTTGTGAAAACCAGAGCACTTGTGGACGCTTTAAAAAATTGGATTGA 2067
FL2        2008 AATAACAATTCTCTTTGTGAAAACCAGAGCACTTGTGGACGCTTTAAAAAATTGGATTGA 2067
SV1         925 AATAACAATTCTCTTTGTGAAAACCAGAGCACTTGTGGACGCTTTAAAAAATTGGATTGA 984
SV2         805 AATAACAATTCTCTTTGTGAAAACCAGAGCACTTGTGGACGCTTTAAAAAATTGGATTGA 864

AF038963.1 2101 AGGAAATCCTAAACTCAGTTTTCTAAAACCTGGCATATTGACTGGACGTGGCAAAACAAA 2160
FL1        2068 AGGAAATCCTAAACTCAGTTTTCTAAAACCTGGCATATTGACTGGACGTGGCAAAACAAA 2127
FL2        2068 AGGAAATCCTAAACTCAGTTTTCTAAAACCTGGCATATTGACTGGACGTGGCAAAACAAA 2127
SV1         985 AGGAAATCCTAAACTCAGTTTTCTAAAACCTGGCATATTGACTGGACGTGGCAAAACAAA 1044
SV2         865 AGGAAATCCTAAACTCAGTTTTCTAAAACCTGGCATATTGACTGGACGTGGCAAAACAAA 924

AF038963.1 2161 TCAGAACACAGGAATGACCCTCCCGGCACAGAAGTGTATATTGGATGCATTCAAAGCCAG 2220
FL1        2128 TCAGAACACAGGAATGACCCTCCCGGCACAGAAGTGTATATTGGATGCATTCAAAGCCAG 2187
FL2        2128 TCAGAACACAGGAATGACCCTCCCGGCACAGAAGTGTATATTGGATGCATTCAAAGCCAG 2187
SV1        1045 TCAGAACACAGGAATGACCCTCCCGGCACAGAAGTGTATATTGGATGCATTCAAAGCCAG 1104
SV2         925 TCAGAACACAGGAATGACCCTCCCGGCACAGAAGTGTATATTGGATGCATTCAAAGCCAG 984

AF038963.1 2221 TGGAGATCACAATATTCTGATTGCCACCTCAGTTGCTGATGAAGGCATTGACATTGCACA 2280
FL1        2188 TGGAGATCACAATATTCTGATTGCCACCTCAGTTGCTGATGAAGGCATTGACATTGCACA 2247
FL2        2188 TGGAGATCACAATATTCTGATTGCCACCTCAGTTGCTGATGAAGGCATTGACATTGCACA 2247
SV1        1105 TGGAGATCACAATATTCTGATTGCCACCTCAGTTGCTGATGAAGGCATTGACATTGCACA 1164
SV2         985 TGGAGATCACAATATTCTGATTGCCACCTCAGTTGCTGATGAAGGCATTGACATTGCACA 1044

AF038963.1 2281 GTGCAATCTTGTCATCCTTTATGAGTATGTGGGCAATGTCATCAAAATGATCCAAACCAG 2340
FL1        2248 GTGCAATCTTGTCATCCTTTATGAGTATGTGGGCAATGTCATCAAAATGATCCAAACCAG 2307
FL2        2248 GTGCAATCTTGTCATCCTTTATGAGTATGTGGGCAATGTCATCAAAATGATCCAAACCAG 2307
SV1        1165 GTGCAATCTTGTCATCCTTTATGAGTATGTGGGCAATGTCATCAAAATGATCCAAACCAG 1224
SV2        1045 GTGCAATCTTGTCATCCTTTATGAGTATGTGGGCAATGTCATCAAAATGATCCAAACCAG 1104

AF038963.1 2341 AGGCAGAGGAAGAGCAAGAGGTAGCAAGTGCTTCCTTCTGACTAGTAATGCTGGTGTAAT 2400
FL1        2308 AGGCAGAGGAAGAGCAAGAGGTAGCAAGTGCTTCCTTCTGACTAGTAATGCTGGTGTAAT 2367
FL2        2308 AGGCAGAGGAAGAGCAAGAGGTAGCAAGTGCTTCCTTCTGACTAGTAATGCTGGTGTAAT 2367
SV1        1225 AGGCAGAGGAAGAGCAAGAGGTAGCAAGTGCTTCCTTCTGACTAGTAATGCTGGTGTAAT 1284
SV2        1105 AGGCAGAGGAAGAGCAAGAGGTAGCAAGTGCTTCCTTCTGACTAGTAATGCTGGTGTAAT 1164

AF038963.1 2401 TGAAAAAGAACAAATAAACATGTACAAAGAAAAAATGATGAATGACTCTATTTTACGCCT 2460
FL1        2368 TGAAAAAGAACAAATAAACATGTACAAAGAAAAAATGATGAATGACTCTATTTTACGCCT 2427
FL2        2368 TGAAAAAGAACAAATAAACATGTACAAAGAAAAAATGATGAATGACTCTATTTTACGCCT 2427
SV1        1285 TGAAAAAGAACAAATAAACATGTACAAAGAAAAAATGATGAATGACTCTATTTTACGCCT 1344
SV2        1165 TGAAAAAGAACAAATAAACATGTACAAAGAAAAAATGATGAATGACTCTATTTTACGCCT 1224

AF038963.1 2461 TCAGACATGGGACGAAGCAGTATTTAGGGAAAAGATTCTGCATATACAGACTCATGAAAA 2520
FL1        2428 TCAGACATGGGACGAAGCAGTATTTAGGGAAAAGATTCTGCATATACAGACTCATGAAAA 2487
FL2        2428 TCAGACATGGGACGAAGCAGTATTTAGGGAAAAGATTCTGCATATACAGACTCATGAAAA 2487
SV1        1345 TCAGACATGGGACGAAGCAGTATTTAGGGAAAAGATTCTGCATATACAGACTCATGAAAA 1404
SV2        1225 TCAGACATGGGACGAAGCAGTATTTAGGGAAAAGATTCTGCATATACAGACTCATGAAAA 1284

AF038963.1 2521 ATTCATCAGAGATAGTCAAGAAAAACCAAAACCTGTCCCTGATAAGGAAAATAAAAAACT 2580
FL1        2488 ATTCATCAGAGATAGTCAAGAAAAACCAAAACCTGTCCCTGATAAGGAAAATAAAAAACT 2547
FL2        2488 ATTCATCAGAGATAGTCAAGAAAAACCAAAACCTGTCCCTGATAAGGAAAATAAAAAACT 2547
SV1        1405 ATTCATCAGAGATAGTCAAGAAAAACCAAAACCTGTCCCTGATAAGGAAAATAAAAAACT 1464
SV2        1285 ATTCATCAGAGATAGTCAAGAAAAACCAAAACCTGTCCCTGATAAGGAAAATAAAAAACT 1344

AF038963.1 2581 GCTCTGCAGAAAGTGCAAAGCCTTGGCATGTTACACAGCTGACGTAAGAGTGATAGAGGA 2640
FL1        2548 GCTCTGCAGAAAGTGCAAAGCCTTGGCATGTTACACAGCTGACGTAAGAGTGATAGAGGA 2607
FL2        2548 GCTCTGCAGAAAGTGCAAAGCCTTGGCATGTTACACAGCTGACGTAAGAGTGATAGAGGA 2607
SV1        1465 GCTCTGCAGAAAGTGCAAAGCCTTGGCATGTTACACAGCTGACGTAAGAGTGATAGAGGA 1524
SV2        1345 GCTCTGCAGAAAGTGCAAAGCCTTGGCATGTTACACAGCTGACGTAAGAGTGATAGAGGA 1404
```

```
AF038963.1 2641 ATGCCATTACACTGTGCTTGGAGATGCTTTTAAGGAATGCTTTGTGAGTAGACCACATCC 2700
FL1        2608 ATGCCATTACACTGTGCTTGGAGATGCTTTTAAGGAATGCTTTGTGAGTAGACCACATCC 2667
FL2        2608 ATGCCATTACACTGTGCTTGGAGATGCTTTTAAGGAATGCTTTGTGAGTAGACCACATCC 2667
SV1        1525 ATGCCATTACACTGTGCTTGGAGATGCTTTTAAGGAATGCTTTGTGAGTAGACCACATCC 1584
SV2        1405 ATGCCATTACACTGTGCTTGGAGATGCTTTTAAGGAATGCTTTGTGAGTAGACCACATCC 1464

AF038963.1 2701 CAAGCCAAAGCAGTTTTCAAGTTTTGAAAAAAGAGCAAAGATATTCTGTGCCCGACAGAA 2760
FL1        2668 CAAGCCAAAGCAGTTTTCAAGTTTTGAAAAAAGAGCAAAGATATTCTGTGCCCGACAGAA 2727
FL2        2668 CAAGCCAAAGCAGTTTTCAAGTTTTGAAAAAAGAGCAAAGATATTCTGTGCCCGACAGAA 2727
SV1        1585 CAAGCCAAAGCAGTTTTCAAGTTTTGAAAAAAGAGCAAAGATATTCTGTGCCCGACAGAA 1644
SV2        1465 CAAGCCAAAGCAGTTTTCAAGTTTTGAAAAAAGAGCAAAGATATTCTGTGCCCGACAGAA 1524

AF038963.1 2761 CTGCAGCCATGACTGGGGAATCCATGTGAAGTACAAGACATTTGAGATTCCAGTTATAAA 2820
FL1        2728 CTGCAGCCATGACTGGGGAATCCATGTGAAGTACAAGACATTTGAGATTCCAGTTATAAA 2787
FL2        2728 CTGCAGCCATGACTGGGGAATCCATGTGAAGTACAAGACATTTGAGATTCCAGTTATAAA 2787
SV1        1645 CTGCAGCCATGACTGGGGAATCCATGTGAAGTACAAGACATTTGAGATTCCAGTTATAAA 1704
SV2        1525 CTGCAGCCATGACTGGGGAATCCATGTGAAGTACAAGACATTTGAGATTCCAGTTATAAA 1584

AF038963.1 2821 AATTGAAAGTTTTGTGGTGGAGGATATTGCAACTGGAGTTCAGACACTGTACTCGAAGTG 2880
FL1        2788 AATTGAAAGTTTTGTGGTGGAGGATATTGCAACTGGAGTTCAGACACTGTACTCGAAGTG 2847
FL2        2788 AATTGAAAGTTTTGTGGTGGAGGATATTGCAACTGGAGTTCAGACACTGTACTCGAAGTG 2847
SV1        1705 AATTGAAAGTTTTGTGGTGGAGGATATTGCAACTGGAGTTCAGACACTGTACTCGAAGTG 1764
SV2        1585 AATTGAAAGTTTTGTGGTGGAGGATATTGCAACTGGAGTTCAGACACTGTACTCGAAGTG 1644

AF038963.1 2881 GAAGGACTTTCATTTTGAGAAGATACCATTTGATCCAGCAGAAATGTCCAAATGATATCA 2940
FL1        2848 GAAGGACTTTCATTTTGAGAAGATACCATTTGATCCAGCAGAAATGTCCAAATGATATCA 2907
FL2        2848 GAAGGACTTTCATTTTGAGAAGATACCATTTGATCCAGCAGAAATGTCCAAATGATATCA 2907
SV1        1765 GAAGGACTTTCATTTTGAGAAGATACCATTTGATCCAGCAGAAATGTCCAAATGATATCA 1824
SV2        1645 GAAGGACTTTCATTTTGAGAAGATACCATTTGATCCAGCAGAAATGTCCAAATGATATCA 1704

AF038963.1 2941 GGTCCTCAATCTTCAGCTACAGGGAATGAGTAACTTTGAGTGGAGAAGAAACAAACATAG 3000
FL1        2908 GGTCCTCAATCTTCAGCTACAGGGAATGAGTAACTTTGAGTGGAGAAGAAACAAACATAG 2967
FL2        2908 GGTCCTCAATCTTCAGCTACAGGGAATGAGTAACTTTGAGTGGAGAAGAAACAAACATAG 2967
SV1        1825 GGTCCTCAATCTTCAGCTACAGGGAATGAGTAACTTTGAGTGGAGAAGAAACAAACATAG 1884
SV2        1705 GGTCCTCAATCTTCAGCTACAGGGAATGAGTAACTTTGAGTGGAGAAGAAACAAACATAG 1764

AF038963.1 3001 TGGGTATAATCATGGATCGCTTGTACCCCTGTGAAAATATATTTTTTAAAAATAAAAAAA 3060
FL1        2968 TGGGTATAATCATGGATCGCTTGTACC--------------------------------- 2994
FL2        2968 TGGGTATAATCATGGATCGCTTGTACC--------------------------------- 2994
SV1        1885 TGGGTATAATCATGGATCGCTTGTACC--------------------------------- 1911
SV2        1765 TGGGTATAATCATGGATCGCTTGTACC--------------------------------- 1791

AF038963.1 3061 AAAAA                                                        3065
FL1        2994 -----                                                        2994
FL2        2994 -----                                                        2994
SV1        1911 -----                                                        1911
SV2        1791 -----                                                        1791
```
