# Supplementary material for: Comparative characterization of flavivirus production in two cell lines: Human hepatoma-derived Huh7.5.1-8 and African green monkey kidney-derived Vero
Source: PLoS One. 2020 Apr 24;15(4):e0232274. doi: 10.1371/journal.pone.0232274 (PMC7182267; doi:10.1371/journal.pone.0232274)

Fig 1C_unadjusted_images

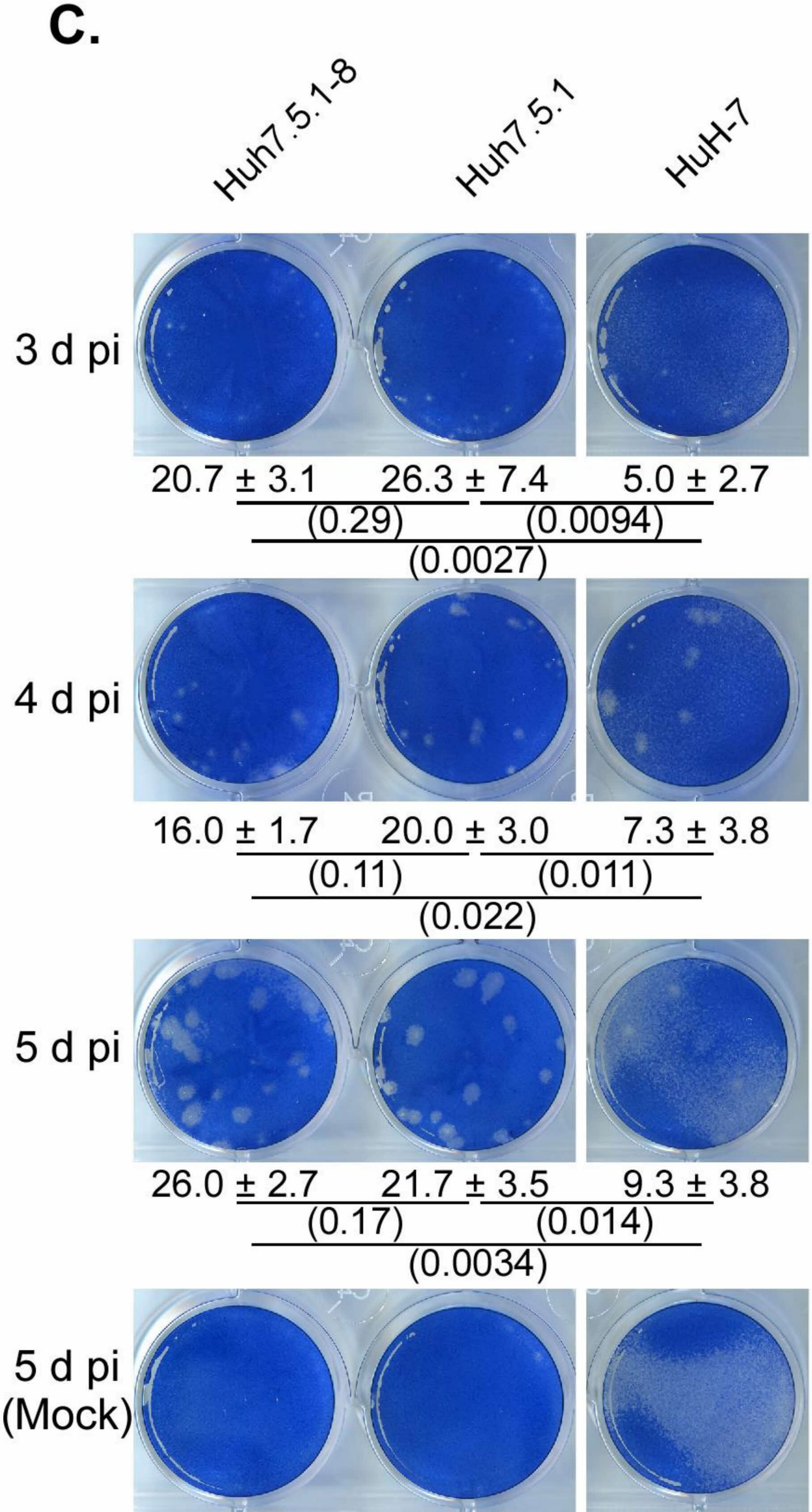

Fig 7_unadjusted_images

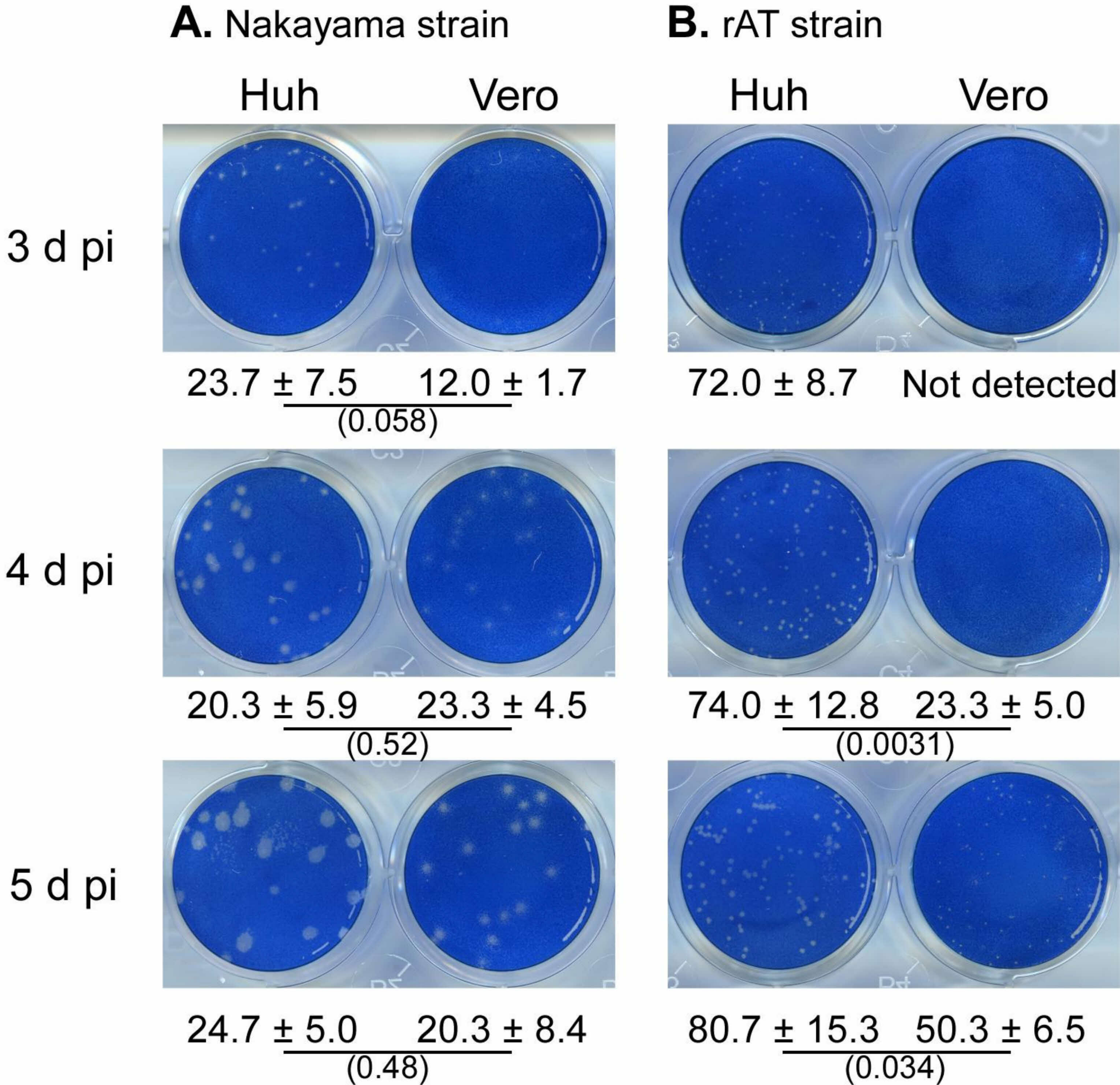

Fig 9_unadjusted_images

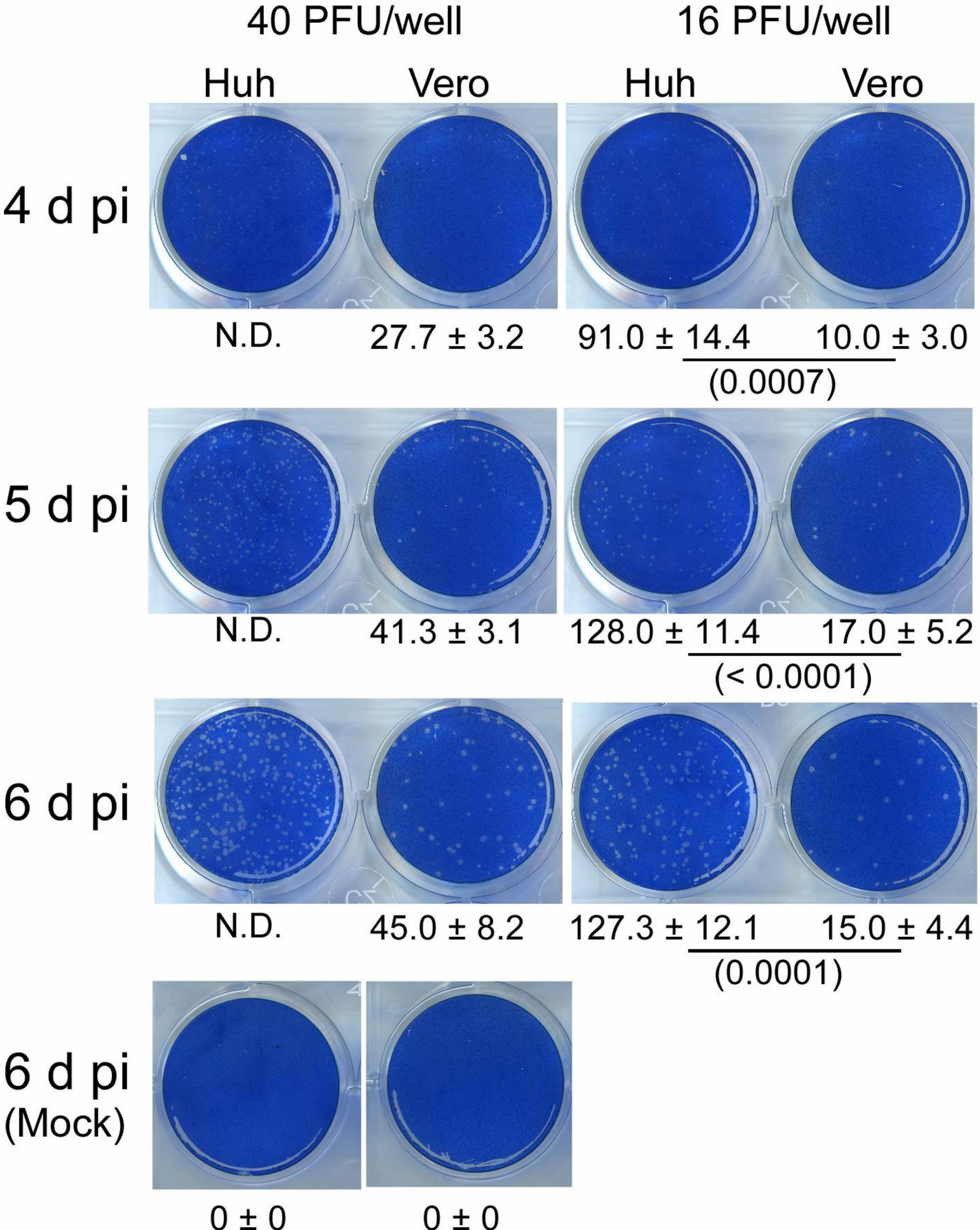

S1B_Fig_unadjusted_images

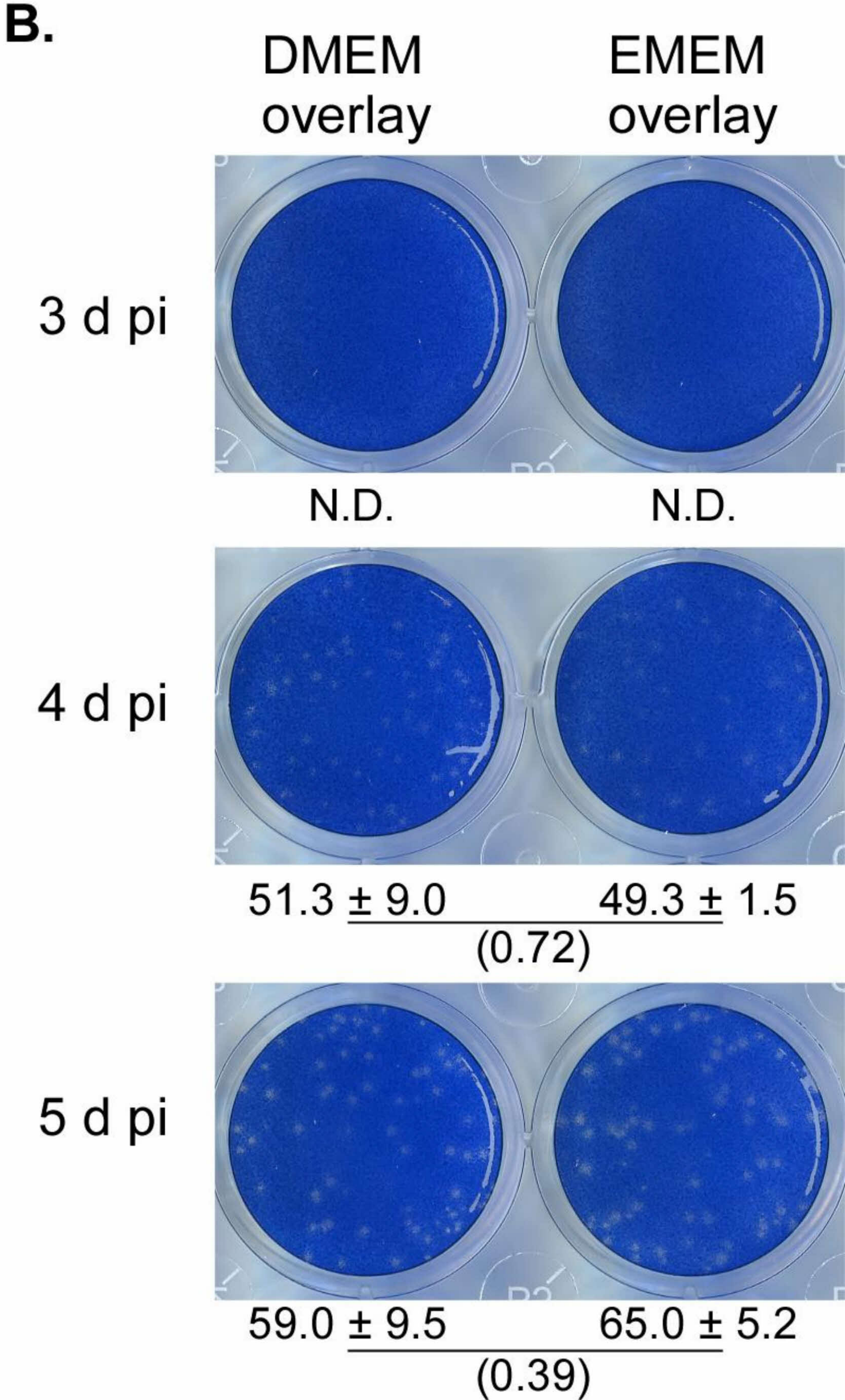

S3_Fig_unadjusted_images

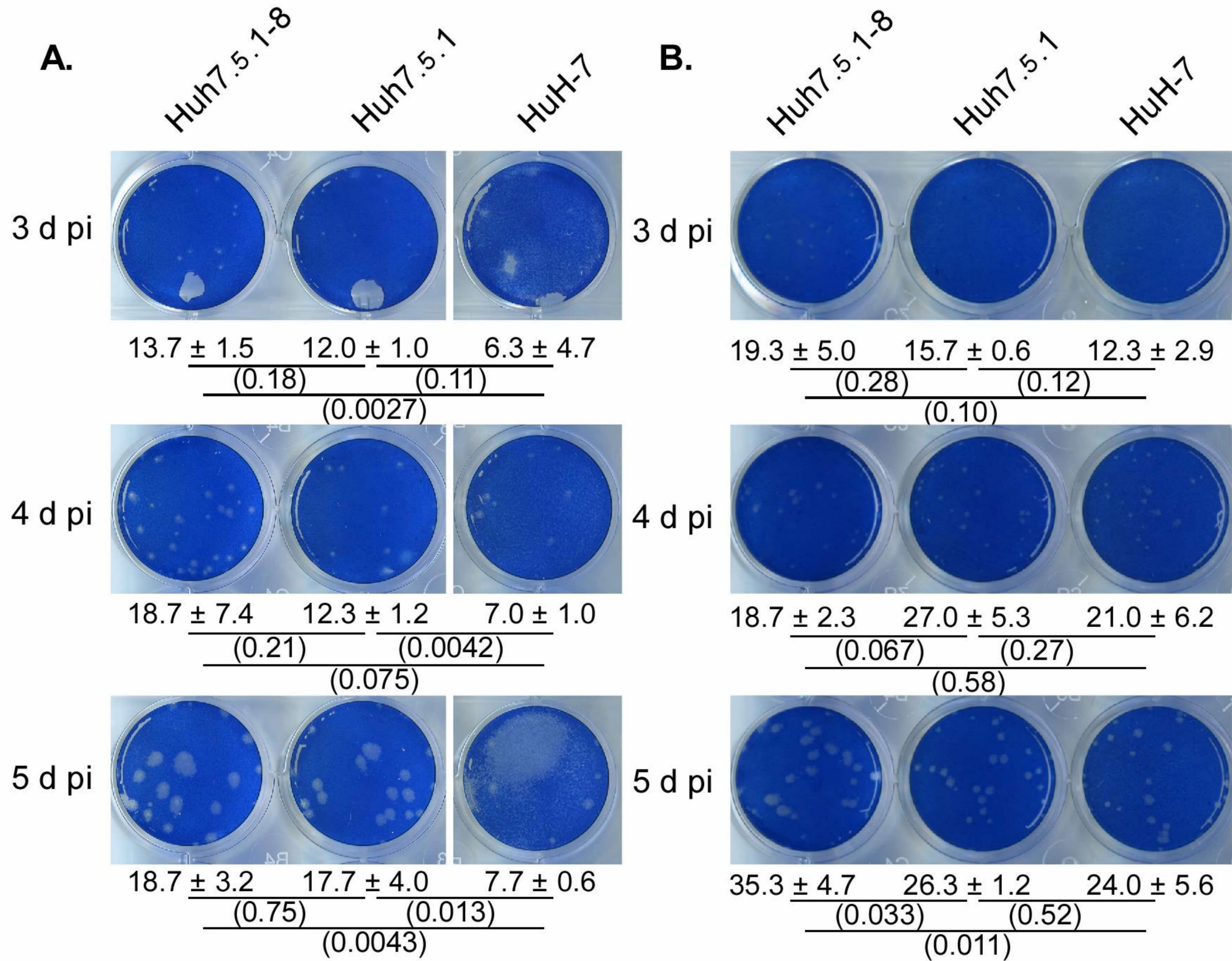

S7_Fig_unadjusted_images

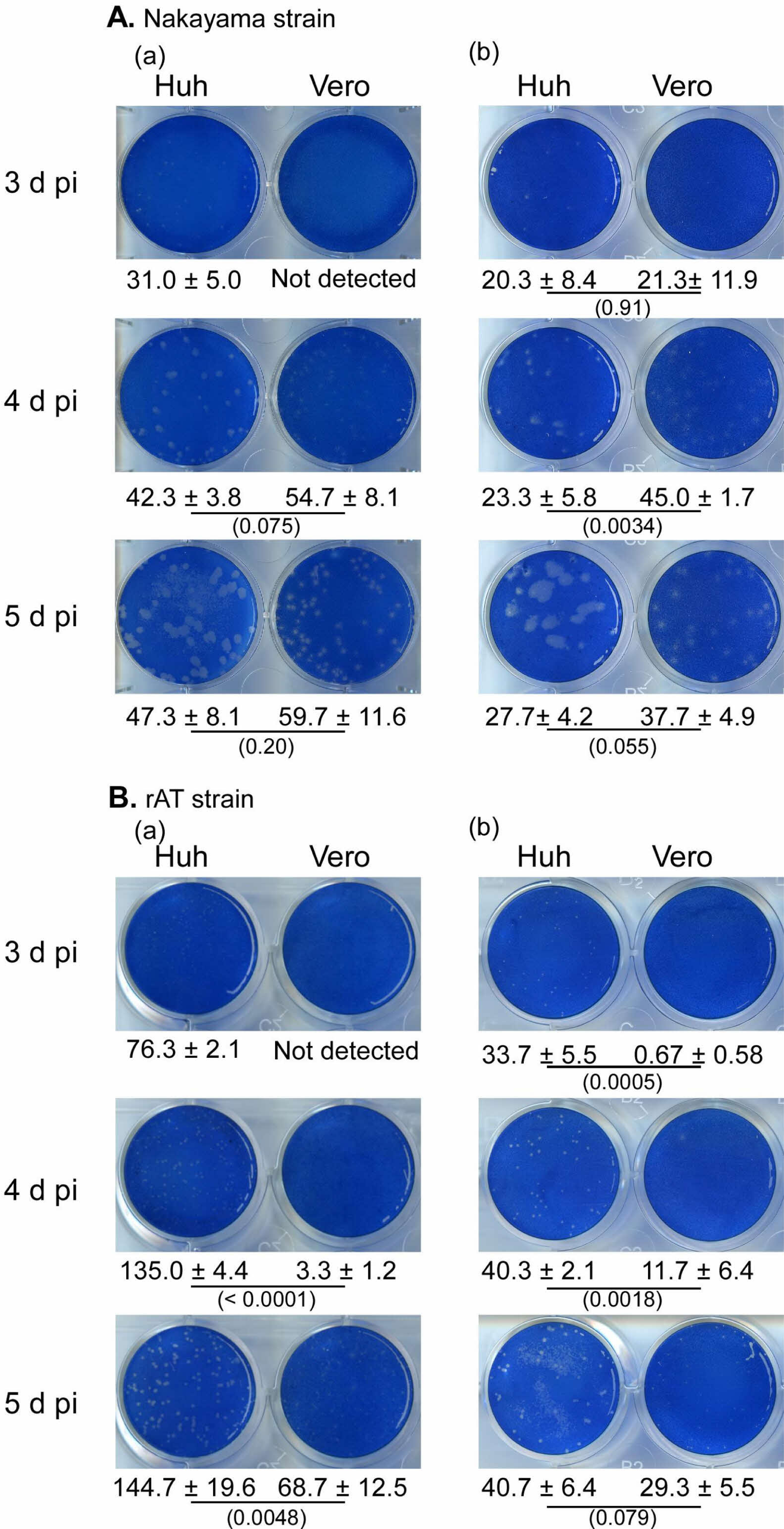

S9_Fig_unadjusted_images

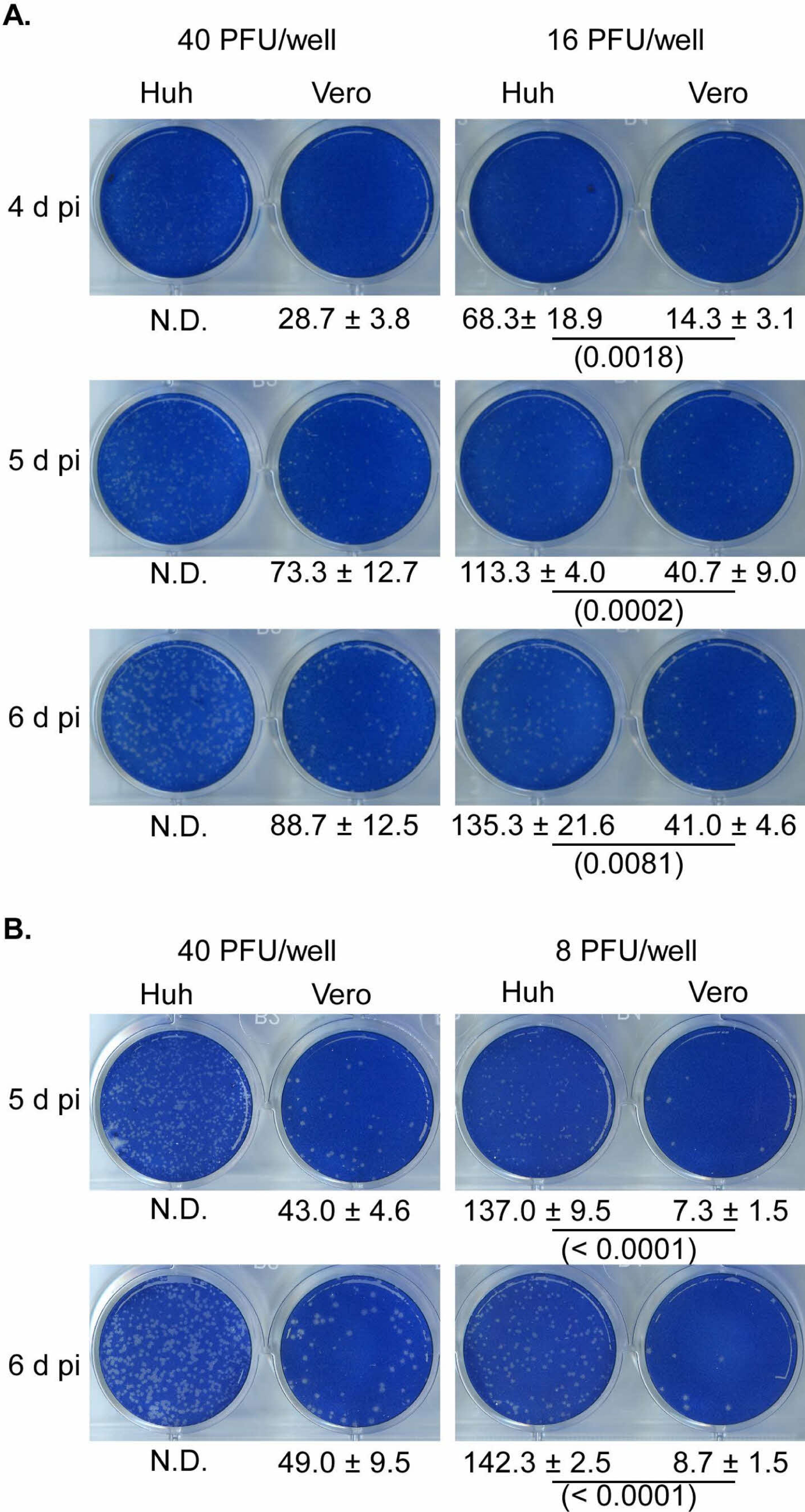

Supplement: S11 Fig — (PDF) [file pone.0232274.s011.pdf]
