# Supplementary material for: Comparative characterization of flavivirus production in two cell lines: Human hepatoma-derived Huh7.5.1-8 and African green monkey kidney-derived Vero
Source: PLoS One. 2020 Apr 24;15(4):e0232274. doi: 10.1371/journal.pone.0232274 (PMC7182267; doi:10.1371/journal.pone.0232274)

Fig 10_raw_image

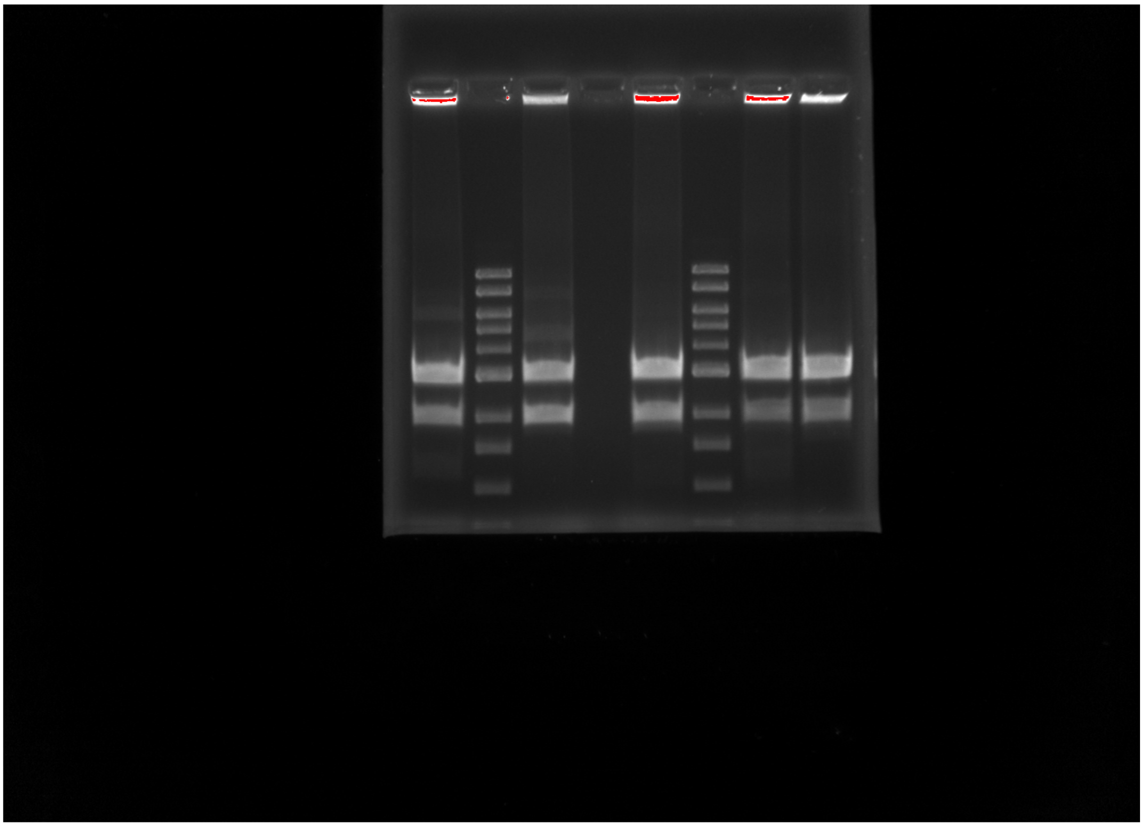

M: MW marker

10, 8, 6, 5, 4, 3, 2, 1.5, 1, 0.5 (kbp)

(from top to bottom)

Acquisition Information

Imager: GelDoc^{TM} EZ (Bio-rad)
Exposure Time (sec): 0.173
(Auto Intense Bands)
Application: Ethidium Bromide
Ref. Bkgd.Time (sec): 10

Flat Field: Applied
Software version: 3.0
Illumination Mode: UV Transillumination
Image Area: X: 150.0 Y: 107.8
Image Pixels: X: 1392 Y: 1000
Pixcel Size (um): X:107.8 Y: 107.8
Data Range (Int): 0-4095

Fig 10_cropped_image

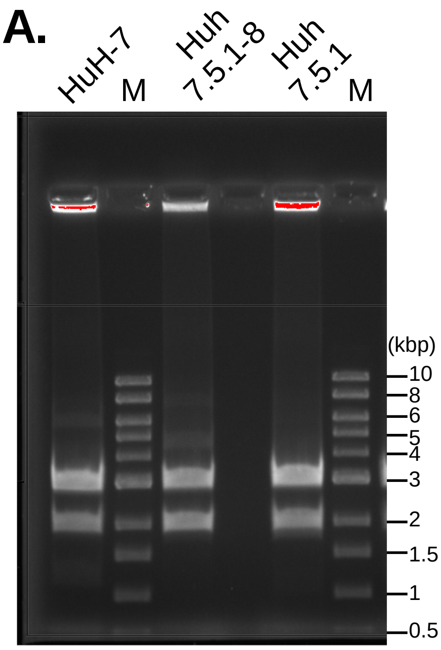

Supplement: S1 Raw Images — (PDF) [file pone.0232274.s012.pdf]
